# A novel real-time PCR assay for the simultaneous detection of the four main causes of bacterial meningitis

**DOI:** 10.1016/j.ijid.2026.108400

**Published:** 2026-03

**Authors:** Kanny Diallo, Tiemele Laurent Simon Amoikon, Kouassi Firmin Missa, Kolotioloman Jérémie Tuo, Odile B. Harrison, Martin C.J. Maiden

**Affiliations:** 1 Centre Suisse de Recherches Scientifiques en Côte d’Ivoire (CSRS), Abidjan, Côte d’Ivoire; 2 Institut Pierre Richet (IPR), Institut national de santé publique (INSP), Bouaké, Côte d’Ivoire; 3 Université Felix Houphouët-Boigny, Abidjan, Côte d’Ivoire; 4 Institut National Polytechnique Felix Houphouët-Boigny, Yamoussoukro, Côte d’Ivoire; 5 Department of Biology, University of Oxford, Oxford, UK; 6 Nuffield Department of Population Health, University of Oxford, Oxford, UK

**Keywords:** Multiplex real-time PCR, Sensitivity, Specificity, PPV, NPV, Efficiency

## Abstract

**Objectives:**

Current multiplex assays cannot detect all four World Health Organization priority pathogens for meningitis diagnosis (*Neisseria meningitidis, Haemophilus influenzae, Streptococcus pneumoniae*, and *Streptococcus agalactiae*). This work developed a novel real-time polymerase chain reaction (PCR) assay capable of simultaneously detecting these pathogens.

**Methods:**

Forty-four DNA samples, including type cultures (NCTC and ATCC), were tested. Specific primers and probes targeting *sodC*, to detect *N. meningitidis, dmsA* for *H. influenzae, SP2020* for *S. pneumoniae*, and *cfb* for *S. agalactiae* were evaluated in monoplex and multiplex. Standard curves were generated, and limits of detection (LLD), slope, intercept, and *R*² were determined. Sensitivity, specificity, and predictive values (positive predictive values/negative predictive values [PPV/NPV]) were assessed for both monoplex and multiplex assays.

**Results:**

Monoplex and multiplex assays showed equivalent performance. Sensitivities were 100% for all targets; specificities ranged 91.7% to 100%, PPVs 72.7% to 100%, and NPVs 100%. The multiplex assay showed high efficiency and consistent amplification for each target gene. LLDs ranged from 24 (*S. pneumoniae*) to 66 (*H. influenzae*) genome copies/µl.

**Conclusion:**

The multiplex PCR assay showed good performance for rapid and accurate detection of meningitis-associated bacteria. This test has applications for improved diagnosis of meningitis in low- and middle-income countries, where laboratory confirmation of major pathogens such as *H. influenzae, S. pneumoniae, N. meningitidis*, and Group B *Streptococcus* remains limited. Field validation with clinical specimens is required before implementation.

## Highlights

• First multiplex PCR for the four WHO priority bacterial meningitis pathogens.
• Assay demonstrates 100% sensitivity for all targets with no performance loss from multiplexing.
• A robust tool for improving meningitis diagnosis, particularly for *Streptococcus agalactiae* (GBS) in LMICs.

## Introduction

Bacterial meningitis is a severe infection of the central nervous system that presents major health risks, including neurological deficits and epilepsy [1]. Among the multiple causes of meningitis, bacterial causes are particularly serious, with *Neisseria meningitidis, Haemophilus influenzae, Streptococcus pneumoniae*, and Group B *Streptococcus agalactiae* (GBS) being the most common and dangerous etiological agents in terms of morbidity and mortality [2]. The incidence of disease caused by these pathogens varies by region, with the highest burden in the African Meningitis Belt [2]. In 2016, this region accounted for over 45% of all worldwide cases [3,4].

While vaccination efforts have significantly reduced the incidence of bacterial meningitis, particularly through the introduction of anti-capsular polysaccharide-conjugate vaccines [[5], [6], [7], [8]], it is unlikely that a vaccine for all capsule variants of these pathogens will be available in the near future [9,10]. For example, to date, there is no licensed vaccine for GBS in part due to the difficulty of conducting efficacy clinical trials in human’s consequent from the low incidence of neonatal disease caused by GBS [11,12]. Therefore, ongoing surveillance is necessary to monitor circulating capsular variants for public health interventions, which requires the development of improved diagnostic tools for the detection of the main pathogens. The World Health Organization (WHO) has identified that poor laboratory capacity to rapidly confirm the presence of epidemic pathogens as a major challenge in controlling meningitis outbreaks [13]. Additionally, timely and accurate diagnosis of bacterial meningitis is crucial to initiate effective treatment and reduce risks of complications, sequelae, and mortality [14].

Bacterial culture remains the gold standard laboratory method for confirming bacterial meningitis; however, it has important limitations, including the time required to obtain definitive results, which is approximately 72 hours. In recent years, molecular methods such as polymerase chain reaction (PCR) have emerged as rapid and effective tools for identifying meningitis-causing bacterial species. Real-time PCR (rtPCR) is the recommended WHO PCR for detecting meningitis bacteria, including the pneumococcus, the meningococcus, and *H. influenzae* type b [15]. Initially, monoplex rtPCR assays were developed, but multiplex rtPCR assays have gained prominence, due to their ability to simultaneously test multiple targets in a single assay, reducing workload, costs, and the amount of sample needed [15]. Although multiplex rtPCR assays detecting *H. influenzae*, meningococci, and pneumococci have been developed [[16], [17], [18], [19]], a limited number of tests that simultaneously detect the four main causes of bacterial meningitis exist. For example, Favaro et al. [20] developed a multi-target rtPCR assay targeting *lytA, ctrA, ompP2, cfb, iap*, and 18S rDNA genes to detect six meningitis-associated microorganisms, including *S. pneumoniae, N. meningitidis, H. influenzae, S. agalactiae, Listeria monocytogenes*, and *Cryptococcus neoformans*.

There are limitations associated with the genetic targets used for the detection of *S. pneumoniae, N. meningitidis*, and *H. influenzae*. For instance, *lytA* homologs may be present in closely related *Streptococcus* species, leading to potential false-positive results [21]. The *ctrA* assay primarily detects encapsulated meningococci, while *ompP2* gene requires high genome copies for detection and exhibits lower sensitivity [15]. In a recent study, we identified alternative genes for the detection of *S. pneumoniae: SP2020* and *psaA; N. meningitidis: sodC* and *porA; H. influenzae: dmsA* (*HAEM1183*) and *hpd; S. agalactiae: cfb* and *sip* [22]. Here, we developed a multiplex rtPCR assay targeting *sodC, dmsA, SP2020*, and *cfb* genes to improve the diagnosis of these pathogens.

## Material and methods

### DNA samples

A total of 44 DNA samples were used for the validation of the multiplex rtPCR assay (Table 1). This included DNA extracts from seven control bacteria obtained from the National Collection of Type Cultures (NCTC). In addition, 28 DNA extracts kindly donated by Dr Mignon du Plessis from the National Institute for Communicable Diseases of South Africa, including four from different control strains and 24 from blood cultures, CSF, pleural fluid, and patient tissue (Table 1), were used. Finally, DNA extracts from *Neisseria lactamica, Neisseria bergeri, N. meningitidis*, and *Moraxella catarrhalis* collected as part of a carriage study conducted in Côte d’Ivoire [23] were used, with species identity confirmed by whole genome sequencing.

**Table 1:** List of species of which DNA extract samples were used for real-time PCR multiplex analysis.

Strains	ID	Clinical source	Country
*Haemophilus influenzae a*	66643	Blood culture	South Africa
*H. influenzae b*	67405	CSF	South Africa
*H. influenzae c*	67657	Blood Culture	South Africa
*H. influenzae d*	65424	Blood culture	South Africa
*H. influenzae e*	61595	Blood culture	South Africa
*H. influenzae f*	67954	CSF	South Africa
*Haemophilus haemolyticus*	QAF GSK	Control strain	South Africa
*Neisseria meningitidis A*	ATCC13077	Control strain	South Africa
*N. meningitidis B*	61370	Blood culture	South Africa
*N. meningitidis C*	61635	Blood culture	South Africa
*N. meningitidis X*	46414	Blood culture	South Africa
*N. meningitidis W*	65322	Blood culture	South Africa
*N. meningitidis Y*	61697	Blood culture	South Africa
*Neisseria lactamica*	ATCC23970	Control strain	South Africa
*Neisseria gonorrhoeae*	WHO	Control strain	South Africa
*Streptococcus agalactiae Ia*	62983	Blood culture	South Africa
*S. agalactiae Ib*	62968	Blood culture	South Africa
*S. agalactiae II*	62378	Tissue	South Africa
*S. agalactiae III*	63709	Blood culture	South Africa
*S. agalactiae IV*	63381	CSF	South Africa
*S. agalactiae V*	63735	Blood culture	South Africa
*Streptococcus pneumoniae 6A*	65954	Pleural fluid	South Africa
*S. pneumoniae 6B*	66773	Blood culture	South Africa
*S. pneumoniae 1*	60105	Blood culture	South Africa
*S. pneumoniae 12F*	67999	Blood culture	South Africa
*S. pneumoniae 14*	67044	Blood culture	South Africa
*S. pneumoniae 19F*	68003	Blood culture	South Africa
*S. pneumoniae 23F*	67951	CSF	South Africa
*N. meningitidis*	04-0005-S3_oro	Oropharyngeal sample	Côte d’Ivoire
*N. meningitidis*	04-0005-S3_sal	Saliva sample	Côte d’Ivoire
*N. lactamica*	04-0036-1	Oropharyngeal sample	Côte d’Ivoire
*N. lactamica*	04-0036-3	Oropharyngeal sample	Côte d’Ivoire
*Neisseria bergeri*	04-0006-3_oro	Oropharyngeal sample	Côte d’Ivoire
*N. bergeri*	04-0020-3_oro	Oropharyngeal sample	Côte d’Ivoire
*N. bergeri*	04-0020-3_sal	Saliva sample	Côte d’Ivoire
*Moraxella catarrhalis*	03-0025-4	Oropharyngeal sample	Côte d’Ivoire
*M. catarrhalis*	03-0028-5	Oropharyngeal sample	Côte d’Ivoire
*H. influenzae*	NCTC 8143	Control strain	United Kingdom
*H. haemolyticus*	NCTC 10659	Control strain	United Kingdom
*Haemophilus aegyptius*	NCTC 8502	Control strain	USA
*N. lactamica*	NCTC 10617	Control strain	USA
*S. agalactiae*	NCTC 8181	Control strain	United Kingdom
*S. pneumoniae*	NCTC 7465	Control strain	USA
*Streptococcus mitis*	NCTC 12261	Control strain	Denmark

### Monoplex rtPCR assay and optimization

rtPCR targeting *sodC, cfb*, and *SP2020* were performed as described previously by Dolan et al. [24], Carrillo-Ávila et al. [25], and Tavares et al. [26], respectively. For *dmsA*, the primers and probes were designed using Primer 3 [27] with default settings and tested as part of a previous study [22]. The primers and probe corresponding to each target (Table 2) were tested with DNA extracts from control strains from their respective organisms (Table 1) using a CFX96 Touch rtPCR Detection system (Bio-Rad) and the TaqMan Gene Expression Master Mix (Applied Biosystems). Monoplex PCR assays were then optimized with a final concentration of 0.5 μM for primers and probes. The assays were subsequently repeated using the DNA extracts from all 44 bacterial isolates (Table 1). Each experiment included no-template controls. The cycling parameters consisted of 2 minutes at 50°C, 10 minutes at 95°C, 45 cycles of 95°C for 15 seconds and 60°C for 1 minute, and a holding stage at 4°C. For the assay, positive specimens were defined as having Ct ≤ 35.

**Table 2:** Primer sequences used for real-time PCR.

Gene	Primer name^a^	5′-3′ nucleotide sequence	References
*cfb*	cfb-F2	GAAACATTGATTGCCCAGC	[37]
	cfb-R2	AGGAAGATTTATCGCACCTG	
	cfb-PB2	Cy3-CCATTTGATAGACGTTCGTGAAGAG-BHQ	
*sodC*	fwd 351	GCACACTTAGGTGATTTACCTGCAT	[23]
	Rev 478	CCACCCGTGTGGATCATAATAGA	
	Pb387	JOE-CATGATGGCACAGCAA-BHQ	
*SP2020*	SP_2020_F	TAAACAGTTTGCCTGTAGTCG	[26]
	SP_2020_R	CCCGGATATCTCTTTCTGGA	
	SP_2020_P	Cy5-AACCTTTGTTCTCTCTCGTGGCAGCTCAA-BHQ	
*dmsA*	HAEM1183_F	TATGGTACGGGAACACTCGG	Paper pending in publication
	HAEM1183_R	ATTTCCCAATGCCCAACCAC	
	HAEM1183_PB	FAM-GTGATTACAGCACCGCACAA-BHQ1	

a All fluorophores and quenchers of probes have been modified from what has been published, with the exception of SP2020 quencher.

### Multiplex rtPCR assay

For each of the primer sets (Table 2), multiplex-rtPCR was performed in a 15 μl reaction volume, containing 7.5 μl of 2 × Master Mix, 0.25 μM of each primer (forward and reverse), 0.25 μM of each probe, template DNA (2 μl), and UltraPure DNase/RNase-Free Distilled Water. The multiplex assay was initially tested using only DNA extracts from *N. meningitidis* ATCC 13077, *S. agalactiae* NCTC 8181, *S. pneumoniae* NCTC 7465, and *H. influenzae* NCTC 8143, before testing the total collection of 44 DNA samples, on a CFX96 Touch rtPCR Detection system (Bio-Rad) under the same conditions as the monoplex assays. The same Ct threshold was used across all experiments.

### Limit of detection (LLD)

DNA extracted from the reference strains: *N. meningitidis* (ATCC 13077), *H. influenzae* (NCTC 8143), *S. agalactiae* (NCTC 8181), and *S. pneumoniae* (NCTC 7465) were serial diluted 5 times, in molecular grade water, at a ratio of 1:10 starting from their initial stock concentrations of 1, 1, 0.17, and 0.24 ng/μl, respectively. The different dilutions were tested using the final conditions of the multiplex assay as presented above.

DNA concentrations were converted from ng/μl to genome copies/μl, using the following formula:

$$\begin{array}{ccc} & & Genome\;copies/\mu l\;\\ & & =\frac{(concentration\;\left(ng/\mu l\right)\;\left(\;\;\times \;\;6.02\;\;\times \;\;10^{23}\;\;\times \;\;10^{-9}\right)}{660\;\times \;whole\;genome\;nucleotide\;number\;} \end{array}$$

[28]

The number of nucleotides in each whole genome was: 2167991 bp for *N. meningitidis* ATCC 13077 (NCBI Reference Sequence: NZ_CP016670); 2110968 bp for *S. pneumoniae* NCTC 7465 (GenBank: LN831051.1); 1890645 bp for *H. influenzae* NCTC 8143 (GenBank: LN831035.1); and 2243750 bp for *S. agalactiae* NCTC 8181 (NCBI Reference Sequence: NZ_GL636070.1).

For each bacterium, a linear regression curve was generated, representing the relationship between the Ct value and the DNA concentration expressed in genome copies/μl.

The LLD therefore, corresponds to the lowest DNA concentration still detectable by the PCR assay.

### Statistical analyses

The sensitivity and specificity, as well as the negative and positive predicted values of the assay, were determined using the following formula:

(a) $$Sensitivity\;\left(Se\right)=\;\frac{a\;\times \;100\;}{a\;+\;c}$$

(b) $$Specificity\;\left(Sp\right)=\;\frac{d\;\times \;100\;}{b\;+\;d}$$

(c) $$Positive\;predictive\;value\;\left(PPV\right)=\;\frac{a\;\times \;100\;}{a\;+\;b}$$

(d) $$Negative\;predictive\;value\;\left(NPV\right)=\;\frac{d\;\times \;100\;}{c\;+\;d}$$

where *a* is true positive samples, *b* is false positive samples, *c* is false negative samples, and *d* is true negative samples.

The standard curves of multiplex real-time PCR were plotted using the ggplot package [29] on R 4.1.0 software [30].

## Results

### Monoplex rtPCR assay performance

The monoplex rtPCR assay detected 7/7 *S. agalactiae* and 7/7 *H. influenzae*, corresponding to a sensitivity of 100% for each (Table 3). Additionally, the assay detected 8/8 *N. meningitidis* and 8/8 *S. pneumoniae*, also corresponding to a sensitivity of 100% (Table 3). Specificity was found to be 100% for assays targeting *S. pneumoniae* and *S. agalactiae*. This was lower for *H. influenzae* (97.3%) and *N. meningitidis* (91.7%) as the assay produced 1 and 3 false positive results, respectively (Table 3).

**Table 3:** Monoplex real-time PCR assays statistics.

Target bacteria	Gene primer set	TP	FP	FN	TN	Se (%)	Sp (%)	PPV (%)	NPV (%)
*Haemophilus influenzae*	*dmsA*	7	1	0	36	100	97.3	87.5	100
*Neisseria meningitidis*	*sodC*	8	3	0	33	100	91.7	72.7	100
*Streptococcus agalactiae*	*Cfb*	7	0	0	37	100	100	100	100
*Streptococcus pneumoniae*	*SP2020*	8	0	0	36	100	100	100	100

CI, confidence interval; FN, false negative; FP, false positive; Se, sensibility; Sp, specificity; TN, true negative; TP, true positive.

The positive predictive values (PPV) were 100% for *S. agalactiae* and *S. pneumoniae*, 87.5% (7/8) for *H. influenzae*, and 72.7% (8/11) for *N. meningitidis*. The negative predictive values (NPV) were 100% for all 4 species (Table 3).

### Performance of the multiplex rtPCR assay

Results showed that the multiplex rtPCR assay successfully detected 7/7 *S. agalactiae* and 7/7 *H. influenzae* (Tables S1 and S3), giving a detection sensitivity of 100% for both pathogens. Additionally, 8/8 *S. pneumoniae* and 8/8 *N. meningitidis* strains were detected (Tables S2 and S4), also giving a sensitivity of 100% (Table 4). The specificity of the assay was 37/37 (100%) for *S. agalactiae*, 36/36 (100%) for *S. pneumoniae*, 36/37 (97.3%) for *H. influenzae*, and 33/36 (91.7%) for *N. meningitidis* (Table 4).

**Table 4:** Sensitivity and specificity of the real-time PCR multiplex assay.

Target pathogen	TP/TP + FN	Se (%)	TN/TN + FP	Sp (%)
*Streptococcus agalactiae* (*cfb)*	7/7	100	37/37	100
*Haemophilus influenzae* (*dmsA*)	7/7	100	36/37	97.3
*Neisseria meningitidis* (*sodC*)	8/8	100	33/36	91.7
*Streptococcus pneumoniae* (*SP2020*)	8/8	100	36/36	100

CI, confidence interval; FN, false negative; FP, false positive; Se, sensibility; Sp, specificity; TN, true negative; TP, true positive.

Furthermore, the PPV and NPV of the multiplex rtPCR assay were determined. The *S. agalactiae* target showed a PPV of 7/7 (100%) and a NPV of 37/37 (100%). The *S. pneumoniae* target showed a PPV of 8/8 (100%) and a NPV of 36/36 (100%) each (Table 5). *H. influenzae* exhibited a PPV of 7/8 (87.5%) and a NPV of 36/36 (100%). The PPV and NPV for *N. meningitidis* were 8/11 (72.7%) and 33/33 (100%), respectively (Table 5).

**Table 5:** Positive and negative predictive values of the real-time PCR multiplex assay.

Target pathogen gene	TP/TP + FP	PPV (%)	TN/TN + FN	NPV (%)
*Streptococcus agalactiae* (*cfb)*	7/7	100	37/37	100
*Haemophilus influenzae* (*dmsA*)	7/8	87.5	36/36	100
*Neisseria meningitidis* (*sodC*)	8/11	72.7	33/33	100
*Streptococcus pneumoniae* (*SP2020*)	8/8	100	36/36	100

CI, confidence interval; FN, false negative; FP, false positive; NPV, negative predictive values; PPV, positive predictive values; TN, true negative; TP, true positive.

### Efficiency and LLD of the assay

**Figure 1:**
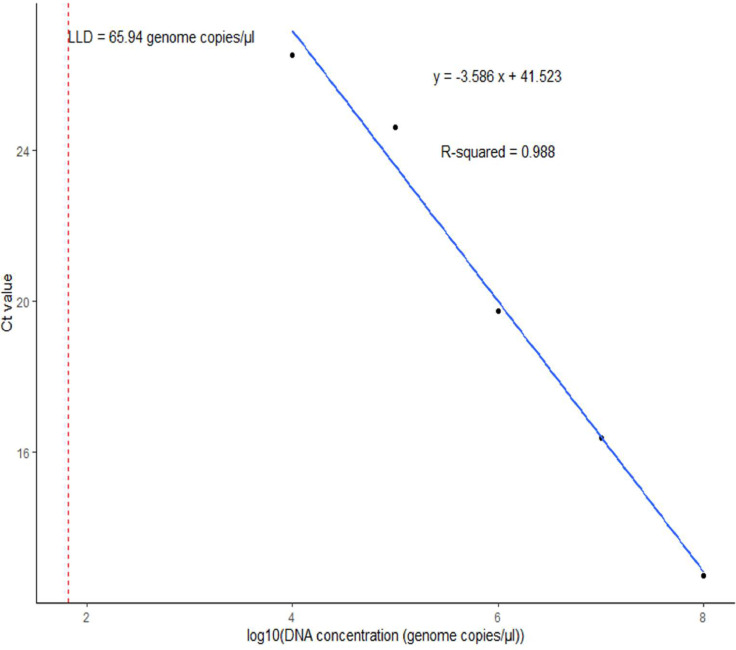
Standard curve of the multiplex real-time PCR assay representing the 10-fold dilution series of DNA of *Haemophilus influenzae.* The graph shows the values of slope, correlation coefficient (*R*^2^), efficiency (*ε*), y-intercept (*y*), and low limit of detection (LLD) for *dmsA.*

**Figure 2:**
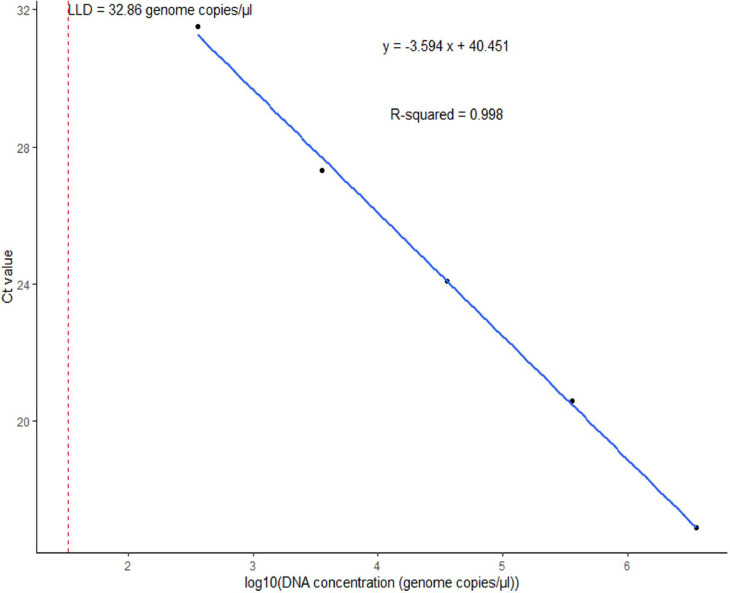
Standard curve of the multiplex real-time PCR assay representing the 10-fold dilution series of DNA of *Neisseria meningitidis*. The graph shows the values of slope, correlation coefficient (*R*^2^), efficiency (*ε*), y-intercept (*y*), and low limit of detection (LLD) for *sodC*.

**Figure 3:**
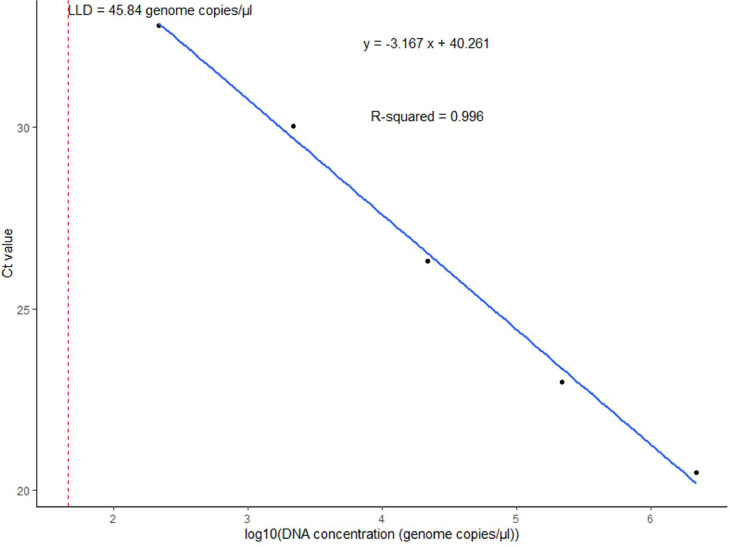
Standard curve of the multiplex real-time PCR assay representing the 10-fold dilution series of DNA of *Streptococcus agalactiae*. The graph shows the values of slope, correlation coefficient (*R*^2^), efficiency (*ε*), y-intercept (*y*), and low limit of detection (LLD) for *cfb.*

**Figure 4:**
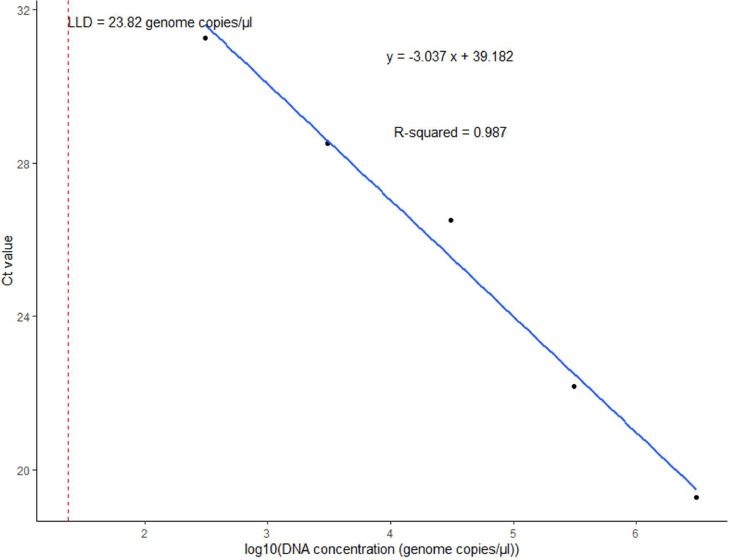
Standard curve of the multiplex real-time PCR assay representing the 10-fold dilution series of DNA of *Streptococcus pneumoniae*. The graph shows the values of slope, correlation coefficient (*R*^2^), efficiency (*ε*), y-intercept (*y*), and low limit of detection (LLD) for *SP2020*.

According to the standard curve derived from the multiplex rtPCR assay of the 10-fold dilution series of crude DNA from control DNA, the Ct limit for *H. influenzae, N. meningitidis, S. agalactiae*, and *S. pneumoniae* was determined to be 35 cycles. This corresponds to an LLD of 66 genome copies/µl for *H. influenzae* (Figure 1), 33 genome copies/µl for *N. meningitidis* (Figure 2), 46 genome copies/µl for *S. agalactiae* (Figure 3), and 24 genome copies/µl for *S. pneumoniae* (Figure 4). Thus, samples with a Ct value <35 were positive and samples >35 were considered negative for the multiplex assay.

For *H. influenzae*, the *dmsA* primers showed a slope (*E*) of −3.586, a correlation coefficient (*R*^2^ of 0.988, and an efficiency (*ε*) of 90.05% (Figure 1). The y-intercept (*y*) of 41.523 represented the Ct value at a DNA concentration of zero. Similarly, for *N. meningitidis*, the *sodC* primers showed *E* = −3.594, *R*^2^ = 0.998, *ε* = 89.78%, and *y* = 40.451 (Figure 2). For *S. agalactiae*, the *cfb* primers showed *E* = −3.167, *R*^2^ = 0.996, *ε* = 106.90%, and *y* = 40.261 (Figure 3). Lastly, for *S. pneumoniae*, the *SP2020* primers showed *E*= −3.037, *R*^2^ = 0.987, *ε* = 113.44%, and *y* = 39.182 (Figure 4).

## Discussion

The use of multiplex PCR assays is advantageous in resource-limited settings, as it reduces the amount of sample and reagents needed and facilitates a more rapid, simultaneous diagnosis of multiple pathogens [20,31]. Here, a multiplex rtPCR assay allowing the detection of 4 WHO priority bacterial meningitis pathogens was developed. The assay demonstrated excellent sensitivity (100%), specificity (91.7%-100%), PPV (72.7%-100%), and NPV (100%) for the detection of *S. agalactiae, H. influenzae, N. meningitidis*, and *S. pneumoniae*, with results similar to those reported previously by Wang et al. [19]. The false-positive results observed for *H. influenzae* and *N. meningitidis* in both monoplex and multiplex rtPCR assays may be attributed to cross-amplification with closely related species, a well-recognized limitation in molecular diagnostics [32]. For *H. influenzae*, commensal species such as *Haemophilus haemolyticus* share close genetic relatedness; although the *dmsA* gene has not been formally reported in this species, the presence of homologous regions or nonspecific primer binding cannot be excluded. Likewise, false-positive amplification for *N. meningitidis* may arise from closely related *Neisseria* species, as evidenced by the amplification of all three *N. bergeri* isolates using the *sodC* primers.

The LLDs obtained for the multiplex assay ranged from 24 to 66 genome copies/µl for *S. pneumoniae, N. meningitidis*, and *H. influenzae*, which is within the range of LLDs of 1-210 genome equivalents per reaction obtained by Wang et al. [19] for the same pathogens using *lytA, ctrA,* and *hpd* as gene targets. In terms of assay efficiency, the multiplex PCR test demonstrated reliable performance. The correlation coefficient (*R*^2^) values close to 1 for all targets (0.98-0.99) indicate a strong linear relationship between the Ct values and the logarithm of DNA concentration. This suggests accurate and reliable amplification of the target DNA. As highlighted by Broeders et al. [33], *R*^2^ should be interpreted alongside other parameters, such as amplification efficiency (*E*) and the slope of the standard curve. The amplification efficiency (*ε*) and the slope of the standard curve should be taken into account to obtain a comprehensive understanding of assay performance [34].

The combination of these parameters provides a more comprehensive understanding of the assay performance and allows for accurate quantification of target concentrations. The efficiency of our assay ranges from 89.78% to 113.44%: this confirms the effectiveness of the test for DNA amplification. Broeders et al. [33] set out criteria for a multiplex test in their guideline; efficiency corresponds to *ε* between 80% and 120% (corresponding to a slope between −3.9 and −2.9) and *R*^2^ ≥ 0.98. The assay developed in this study demonstrated its efficiency, but has been tested with laboratory-grown bacteria only. Additional tests with real patient samples, which were beyond the scope of this study, are required to further evaluate the assay.

In this study, we used *sodC, SP2020, cfb* and *dmsA* as genetic targets. The *sodC* gene, recommended by WHO/CDC for its high specificity and sensitivity in detecting *N. meningitidis*, encodes Cu-Zn superoxide dismutase, a ubiquitous protein in the species that is less prone to antigenic variation, making it a reliable molecular target [24]. The SP2020 gene is a putative GntR-family transcriptional regulator, part of the core genome of *S. pneumoniae*, and is nearly universally present in pneumococci but absent in nonpneumococcal streptococci [26]. The *cfb* gene encodes the extracellular pore-forming toxin (CAMP factor). This gene has been shown to be an effective target for GBS detection [25]. The *dmsA* gene is part of the core genome of *H. influenzae* and plays a role in its fitness [35]. It has shown to be an effective marker for identifying *H. influenzae* [22]. While the primers for *sodC, SP2020*, and *cfb* were adopted from published studies without modification, the *dmsA* primers and probe were designed and validated by *in silico* and *in vitro* analysis, addressing limitations in detecting *H. influenzae* reported with previous assays.

Several multiplex assays have been developed to detect bacterial pathogens associated with meningitis, including those targeting *ctrA, bexA*, and *ply* [36]; *ctrA, lytA*, and *hpd* [14,19]; *lytA, ctrA, ompP2*, and *cfb* [20]; and *sodC, lytA*, and *hpd* [18]. However, some of the targets used have limitations. For example, while *bexA* is effective for detecting encapsulated *H. influenzae*, it fails to identify nontypeable strains. Similarly, assays targeting *ply* for *S. pneumoniae* show reduced specificity compared to more robust markers like *lytA*. Although *lytA* is recommended by WHO/CDC and commonly used for *S. pneumoniae* surveillance, homologs in related *Streptococcus* species could increase false positives [[37], [38], [39]]. Additionally, assays targeting *ctrA* have produced false negatives for invasive isolates with *ctrA* alleles exhibiting low sequence identity to published references [40]. For GBS detection, the WHO does not recommend a specific target. Currently, this pathogen is not systematically tested in routine meningitis surveillance in most countries. Therefore, the multiplex assay proposed in this study enables simultaneous testing for the most common pathogens in a single test.

In contrast, a recent study [22] demonstrated that the genetic targets used in this assay offered superior performance, with metrics exceeding 90% across sensitivity, specificity, PPV, and NPV. Although our assay demonstrated strong performance, these results are based on the evaluation of 44 strains. This test allows rapid detection of the agents responsible for meningitis, but it does not provide information on their susceptibility patterns. Therefore, culture remains essential to guide appropriate clinical management. While this dataset supports the reliability of the assay, the relatively small number of strains analyzed is a limit of the study. Further validation with a larger and more diverse strain collection is necessary to further confirm the suitability of this diagnostic test.

In conclusion, the multiplex assay developed as part of this study demonstrated high performance and efficacy. Although further validation is required, the test has the capacity to detect four of the most common pathogens involved in meningitis. The implementation of such test in low- and middle-income countries is important to improve diagnostic capabilities and determine the real burden of these pathogens to better inform public health strategies.

## Author contributions

KD: Conceptualization, investigation, methodology, project administration, resources, supervision, validation, writing—review and editing. TLSA: Conceptualization, formal analysis, investigation, methodology, visualization, writing—original draft, writing—review and editing. KJT and KFM: Formal analysis, investigation, writing—original draft. OBH and MCJM: Conceptualization, data curation, funding acquisition, methodology, project administration, resources, validation, writing—review and editing.

## Declaration of generative AI and AI-assisted technologies in the writing process

The authors declare that no Generative AI was used in the creation of this manuscript.

## Ethical approval

Ethical approval was not required for this study, as it did not involve human participants or animal subjects.

## Funding

This research received funding from the Department of Health and Social Care through UK Aid as part of the UK Vaccine Network, managed by NIHR. The opinions expressed in this publication are those of the author(s) and do not necessarily reflect the views of the Department of Health and Social Care. Dr. Diallo was supported by a Crick African Network Fellowship and the DELTAS Africa Initiative (Afrique One-ASPIRE/DEL-15-008).

## Declaration of competing interests

The authors declare that they have no known competing financial interests or personal relationships that could have appeared to influence the work reported in this article.

## Appendix Supplementary materials

Image, application 1
