# Supplementary material for: A novel real-time PCR assay for the simultaneous detection of the four main causes of bacterial meningitis
Source: Int J Infect Dis. 2026 Mar;164:None. doi: 10.1016/j.ijid.2026.108400 (PMC12979003; doi:10.1016/j.ijid.2026.108400)
Supplement: Supplementary file 1 [file mmc1.docx]

**Supplementary files**

Table S1: Ct values of multiplex assay targeting *cfb*.

| Well | Fluor | Target | DNA sample from | Ct value |
| --- | --- | --- | --- | --- |
| A01 | Cal Red 610 | *Cfb* | *N. meningitis* ATCC13077 | - |
| B01 | Cal Red 610 | *Cfb* | *N. meningitis* 61370 | - |
| C01 | Cal Red 610 | *Cfb* | *N. meningitis* 61635 | - |
| D01 | Cal Red 610 | *Cfb* | *N. meningitis* 65322 | - |
| E01 | Cal Red 610 | *Cfb* | *N. meningitis* 46414 | - |
| F01 | Cal Red 610 | *Cfb* | *N. meningitis* 61697 | - |
| H01 | Cal Red 610 | *Cfb* | *N. meningitis* 04-0005-S3 Sal | - |
| A02 | Cal Red 610 | *Cfb* | *N. meningitis* 04-0005-S3 Oro | - |
| B02 | Cal Red 610 | *Cfb* | *N. lactamica* 04-0036-1 | - |
| C02 | Cal Red 610 | *Cfb* | *N. lactamica* 04-0036-3 | - |
| D02 | Cal Red 610 | *Cfb* | *Neisseria* *bergeri* 04-0020-3 Oro | - |
| E02 | Cal Red 610 | *Cfb* | *N. bergeri* 04-0006-3 | - |
| F02 | Cal Red 610 | *Cfb* | *N.* *bergeri* 04-0020-3 Sal | - |
| G02 | Cal Red 610 | *Cfb* | *N. lactamica* ATCC23970 | - |
| H02 | Cal Red 610 | *Cfb* | *N. lactamica* NCTC 10617 | - |
| A03 | Cal Red 610 | *Cfb* | *N. gonorrhée* WHO | - |
| B03 | Cal Red 610 | *Cfb* | *S. agalactiae* Ia 62983 | 25.09 |
| C03 | Cal Red 610 | *Cfb* | *S. agalactiae* Ib 62968 | 23.40 |
| D03 | Cal Red 610 | *Cfb* | *S. agalactiae* II 62378 | 20.72 |
| E03 | Cal Red 610 | *Cfb* | *S. agalactiae* III 63709 | 20.36 |
| F03 | Cal Red 610 | *Cfb* | *S. agalactiae* IV 63381 | 22.07 |
| G03 | Cal Red 610 | *Cfb* | *S. agalactiae* V 63735 | 23.23 |
| H03 | Cal Red 610 | *Cfb* | *S. agalactiae* NCTC 8181 | 21.11 |
| A04 | Cal Red 610 | *Cfb* | *S. pneumoniae* 6A 65954 | - |
| B04 | Cal Red 610 | *Cfb* | *S. pneumoniae* 6B 66773 | - |
| C04 | Cal Red 610 | *Cfb* | *S. pneumoniae* 1 60105 | - |
| D04 | Cal Red 610 | *Cfb* | *S. pneumoniae* 12F 67999 | - |
| E04 | Cal Red 610 | *Cfb* | *S. pneumoniae* 14 67044 | - |
| F04 | Cal Red 610 | *Cfb* | *S. pneumoniae* 19F 68003 | - |
| G04 | Cal Red 610 | *Cfb* | *S. pneumoniae* 23F 67951 | - |
| H04 | Cal Red 610 | *Cfb* | *S. pneumoniae* NCTC 7465 | - |
| A05 | Cal Red 610 | *cfb* | *S. mitis* NCTC 12261 | - |
| B05 | Cal Red 610 | *cfb* | *H. influenzae* a 66643 | - |
| C05 | Cal Red 610 | *cfb* | *H. influenzae* b 67405 | - |
| D05 | Cal Red 610 | *cfb* | *H. influenzae* c 67657 | - |
| E05 | Cal Red 610 | *cfb* | *H. influenzae* c 65424 | - |
| F05 | Cal Red 610 | *cfb* | *H. influenzae* e 61595 | - |
| G05 | Cal Red 610 | *cfb* | *H. influenzae* f 67954 | - |
| H05 | Cal Red 610 | *cfb* | *H. influenzae* NCTC 8143 | - |
| A06 | Cal Red 610 | *cfb* | *H. haemolyticus* NCTC 10659 | - |
| B06 | Cal Red 610 | *cfb* | *N. haemolyticus* QAF GSK | - |
| C06 | Cal Red 610 | *cfb* | *H. aegyptius* NCTC 8502 | - |
| D06 | Cal Red 610 | *cfb* | *Moraxella catharalis* 03-0025-4 | - |
| E06 | Cal Red 610 | *cfb* | *M. catharalis* 03-0028-5 | - |
| F06 | Cal Red 610 | *cfb* | Water, Molecular Biology Grade | - |
| The assay was performed with a panel of 44 DNA samples and a no template control (Water, Molecular Biology Grade); Ct <35: positive result; Ct ≥ 35: negative result; -: negative result with Ct >50; NCTC, ATCC strains, *N. gonorrhée* WHO and *H. haemolyticus* QAF GSK were control strains, Oro means that the strain was isolated from oropharyngeal sample, Sal means that the strain was isolated from saliva sample. | | | | |

Table S2: Ct values of multiplex assay targeting *SP2020*

| Well | Fluor | Target | DNA sample from | Ct value |
| --- | --- | --- | --- | --- |
| A01 | Cy5 | *SP2020* | *N. meningitis* ATCC13077 | - |
| B01 | Cy5 | *SP2020* | *N. meningitis* 61370 | - |
| C01 | Cy5 | *SP2020* | *N. meningitis* 61635 | - |
| D01 | Cy5 | *SP2020* | *N. meningitis* 65322 | - |
| E01 | Cy5 | *SP2020* | *N. meningitis* 46414 | - |
| F01 | Cy5 | *SP2020* | *N. meningitis* 61697 | - |
| H01 | Cy5 | *SP2020* | *N. meningitis* 04-0005-S3 Sal | - |
| A02 | Cy5 | *SP2020* | *N. meningitis* 04-0005-S3 Oro | - |
| B02 | Cy5 | *SP2020* | *N. lactamica* 04-0036-1 | - |
| C02 | Cy5 | *SP2020* | *N. lactamica* 04-0036-3 | - |
| D02 | Cy5 | *SP2020* | *Neisseria* *bergeri* 04-0020-3 Oro | - |
| E02 | Cy5 | *SP2020* | *N. bergeri* 04-0006-3 | - |
| F02 | Cy5 | *SP2020* | *N.* *bergeri* 04-0020-3 Sal | - |
| G02 | Cy5 | *SP2020* | *N. lactamica* ATCC23970 | - |
| H02 | Cy5 | *SP2020* | *N. lactamica* NCTC 10617 | - |
| A03 | Cy5 | *SP2020* | *N. gonorrhée* WHO | 45.04 |
| B03 | Cy5 | *SP2020* | *S. agalactiae* Ia 62983 | - |
| C03 | Cy5 | *SP2020* | *S. agalactiae* Ib 62968 | - |
| D03 | Cy5 | *SP2020* | *S. agalactiae* II 62378 | - |
| E03 | Cy5 | *SP2020* | *S. agalactiae* III 63709 | - |
| F03 | Cy5 | *SP2020* | *S. agalactiae* IV 63381 | - |
| G03 | Cy5 | *SP2020* | *S. agalactiae* V 63735 | - |
| H03 | Cy5 | *SP2020* | *S. agalactiae* NCTC 8181 | - |
| A04 | Cy5 | *SP2020* | *S. pneumoniae* 6A 65954 | 29.03 |
| B04 | Cy5 | *SP2020* | *S. pneumoniae* 6B 66773 | 24.78 |
| C04 | Cy5 | *SP2020* | *S. pneumoniae* 1 60105 | 22.02 |
| D04 | Cy5 | *SP2020* | *S. pneumoniae* 12F 67999 | 20.54 |
| E04 | Cy5 | *SP2020* | *S. pneumoniae* 14 67044 | 18.75 |
| F04 | Cy5 | *SP2020* | *S. pneumoniae* 19F 68003 | 21.62 |
| G04 | Cy5 | *SP2020* | *S. pneumoniae* 23F 67951 | 21.00 |
| H04 | Cy5 | *SP2020* | *S. pneumoniae* NCTC 7465 | 19.92 |
| A05 | Cy5 | *SP2020* | *S. mitis* NCTC 12261 | - |
| B05 | Cy5 | *SP2020* | *H. influenzae* a 66643 | - |
| C05 | Cy5 | *SP2020* | *H. influenzae* b 67405 | - |
| D05 | Cy5 | *SP2020* | *H. influenzae* c 67657 | - |
| E05 | Cy5 | *SP2020* | *H. influenzae* c 65424 | - |
| F05 | Cy5 | *SP2020* | *H. influenzae* e 61595 | - |
| G05 | Cy5 | *SP2020* | *H. influenzae* f 67954 | - |
| H05 | Cy5 | *SP2020* | *H. influenzae* NCTC 8143 | - |
| A06 | Cy5 | *SP2020* | *H. haemolyticus* NCTC 10659 | - |
| B06 | Cy5 | *SP2020* | *N. haemolyticus* QAF GSK | - |
| C06 | Cy5 | *SP2020* | *H. aegyptius* NCTC 8502 | - |
| D06 | Cy5 | *SP2020* | *Moraxella catharalis* 03-0025-4 | - |
| E06 | Cy5 | *SP2020* | *M. catharalis* 03-0028-5 | - |
| F06 | Cy5 | *SP2020* | Water, Molecular Biology Grade | - |
| The assay was performed with a panel of 44 DNA samples and a no template control (Water, Molecular Biology Grade); Ct <35: positive result; Ct ≥ 35: negative result; -: negative result with Ct >50; NCTC, ATCC strains, *N. gonorrhée* WHO and *H. haemolyticus* QAF GSK were control strains, Oro means that the strain was isolated from oropharyngeal sample, Sal means that the strain was isolated from saliva sample. | | | | |

Table S3: Ct values of multiplex assay targeting *dmsA*

| Well | Fluor | Target | DNA sample from | Ct value |
| --- | --- | --- | --- | --- |
| A01 | FAM | *dmsA* | *N. meningitis* ATCC13077 | - |
| B01 | FAM | *dmsA* | *N. meningitis* 61370 | - |
| C01 | FAM | *dmsA* | *N. meningitis* 61635 | - |
| D01 | FAM | *dmsA* | *N. meningitis* 65322 | - |
| E01 | FAM | *dmsA* | *N. meningitis* 46414 | - |
| F01 | FAM | *dmsA* | *N. meningitis* 61697 | - |
| H01 | FAM | *dmsA* | *N. meningitis* 04-0005-S3 Sal | - |
| A02 | FAM | *dmsA* | *N. meningitis* 04-0005-S3 Oro | - |
| B02 | FAM | *dmsA* | *N. lactamica* 04-0036-1 | - |
| C02 | FAM | *dmsA* | *N. lactamica* 04-0036-3 | - |
| D02 | FAM | *dmsA* | *Neisseria* *bergeri* 04-0020-3 Oro | - |
| E02 | FAM | *dmsA* | *N. bergeri* 04-0006-3 | - |
| F02 | FAM | *dmsA* | *N.* *bergeri* 04-0020-3 Sal | - |
| G02 | FAM | *dmsA* | *N. lactamica* ATCC23970 | 41.32 |
| H02 | FAM | *dmsA* | *N. lactamica* NCTC 10617 | 37.77 |
| A03 | FAM | *dmsA* | *N. gonorrhée* WHO | - |
| B03 | FAM | *dmsA* | *S. agalactiae* Ia 62983 | - |
| C03 | FAM | *dmsA* | *S. agalactiae* Ib 62968 | - |
| D03 | FAM | *dmsA* | *S. agalactiae* II 62378 | - |
| E03 | FAM | *dmsA* | *S. agalactiae* III 63709 | - |
| F03 | FAM | *dmsA* | *S. agalactiae* IV 63381 | - |
| G03 | FAM | *dmsA* | *S. agalactiae* V 63735 | - |
| H03 | FAM | *dmsA* | *S. agalactiae* NCTC 8181 | - |
| A04 | FAM | *dmsA* | *S. pneumoniae* 6A 65954 | - |
| B04 | FAM | *dmsA* | *S. pneumoniae* 6B 66773 | - |
| C04 | FAM | *dmsA* | *S. pneumoniae* 1 60105 | - |
| D04 | FAM | *dmsA* | *S. pneumoniae* 12F 67999 | - |
| E04 | FAM | *dmsA* | *S. pneumoniae* 14 67044 | - |
| F04 | FAM | *dmsA* | *S. pneumoniae* 19F 68003 | - |
| G04 | FAM | *dmsA* | *S. pneumoniae* 23F 67951 | - |
| H04 | FAM | *dmsA* | *S. pneumoniae* NCTC 7465 | - |
| A05 | FAM | *dmsA* | *S. mitis* NCTC 12261 | - |
| B05 | FAM | *dmsA* | *H. influenzae* a 66643 | 24.95 |
| C05 | FAM | *dmsA* | *H. influenzae* b 67405 | 28.10 |
| D05 | FAM | *dmsA* | *H. influenzae* c 67657 | 26.10 |
| E05 | FAM | *dmsA* | *H. influenzae* c 65424 | 28.03 |
| F05 | FAM | *dmsA* | *H. influenzae* e 61595 | 27.70 |
| G05 | FAM | *dmsA* | *H. influenzae* f 67954 | 27.06 |
| H05 | FAM | *dmsA* | *H. influenzae* NCTC 8143 | 11.74 |
| A06 | FAM | *dmsA* | *H. haemolyticus* NCTC 10659 | 24.67 |
| B06 | FAM | *dmsA* | *N. haemolyticus* QAF GSK | - |
| C06 | FAM | *dmsA* | *H. aegyptius* NCTC 8502 | 43.32 |
| D06 | FAM | *dmsA* | *Moraxella catharalis* 03-0025-4 | - |
| E06 | FAM | *dmsA* | *M. catharalis* 03-0028-5 | - |
| F06 | FAM | *dmsA* | Water, Molecular Biology Grade | - |
| The assay was performed with a panel of 44 DNA samples and a no template control (Water, Molecular Biology Grade); Ct <35: positive result; Ct ≥ 35: negative result; -: negative result with Ct >50; NCTC, ATCC strains, *N. gonorrhée* WHO and *H. haemolyticus* QAF GSK were control strains, Oro means that the strain was isolated from oropharyngeal sample, Sal means that the strain was isolated from saliva sample. | | | | |

Table S4: Ct values of multiplex assay targeting *sodC*

| Well | Fluor | Target | DNA sample from | Ct value |
| --- | --- | --- | --- | --- |
| A01 | HEX | *sodC* | *N. meningitis* ATCC13077 | 22.79 |
| B01 | HEX | *sodC* | *N. meningitis* 61370 | 22.08 |
| C01 | HEX | *sodC* | *N. meningitis* 61635 | 22.29 |
| D01 | HEX | *sodC* | *N. meningitis* 65322 | 20.71 |
| E01 | HEX | *sodC* | *N. meningitis* 46414 | 19.25 |
| F01 | HEX | *sodC* | *N. meningitis* 61697 | 20.48 |
| H01 | HEX | *sodC* | *N. meningitis* 04-0005-S3 Sal | 17.97 |
| A02 | HEX | *sodC* | *N. meningitis* 04-0005-S3 Oro | 16.92 |
| B02 | HEX | *sodC* | *N. lactamica* 04-0036-1 | 37.74 |
| C02 | HEX | *sodC* | *N. lactamica* 04-0036-3 | 37.51 |
| D02 | HEX | *sodC* | *Neisseria* *bergeri* 04-0020-3 Oro | 18.46 |
| E02 | HEX | *sodC* | *N. bergeri* 04-0006-3 | 19.32 |
| F02 | HEX | *sodC* | *N.* *bergeri* 04-0020-3 Sal | 33.87 |
| G02 | HEX | *sodC* | *N. lactamica* ATCC23970 | 35.44 |
| H02 | HEX | *sodC* | *N. lactamica* NCTC 10617 | - |
| A03 | HEX | *sodC* | *N. gonorrhée* WHO | - |
| B03 | HEX | *sodC* | *S. agalactiae* Ia 62983 | - |
| C03 | HEX | *sodC* | *S. agalactiae* Ib 62968 | - |
| D03 | HEX | *sodC* | *S. agalactiae* II 62378 | 41.49 |
| E03 | HEX | *sodC* | *S. agalactiae* III 63709 | - |
| F03 | HEX | *sodC* | *S. agalactiae* IV 63381 | 42.55 |
| G03 | HEX | *sodC* | *S. agalactiae* V 63735 | 42.02 |
| H03 | HEX | *sodC* | *S. agalactiae* NCTC 8181 | - |
| A04 | HEX | *sodC* | *S. pneumoniae* 6A 65954 | - |
| B04 | HEX | *sodC* | *S. pneumoniae* 6B 66773 | - |
| C04 | HEX | *sodC* | *S. pneumoniae* 1 60105 | - |
| D04 | HEX | *sodC* | *S. pneumoniae* 12F 67999 | 43.61 |
| E04 | HEX | *sodC* | *S. pneumoniae* 14 67044 | - |
| F04 | HEX | *sodC* | *S. pneumoniae* 19F 68003 | - |
| G04 | HEX | *sodC* | *S. pneumoniae* 23F 67951 | - |
| H04 | HEX | *sodC* | *S. pneumoniae* NCTC 7465 | - |
| A05 | HEX | *sodC* | *S. mitis* NCTC 12261 | - |
| B05 | HEX | *sodC* | *H. influenzae* a 66643 | - |
| C05 | HEX | *sodC* | *H. influenzae* b 67405 | - |
| D05 | HEX | *sodC* | *H. influenzae* c 67657 | - |
| E05 | HEX | *sodC* | *H. influenzae* c 65424 | - |
| F05 | HEX | *sodC* | *H. influenzae* e 61595 | - |
| G05 | HEX | *sodC* | *H. influenzae* f 67954 | - |
| H05 | HEX | *sodC* | *H. influenzae* NCTC 8143 | - |
| A06 | HEX | *sodC* | *H. haemolyticus* NCTC 10659 | - |
| B06 | HEX | *sodC* | *N. haemolyticus* QAF GSK | 43.38 |
| C06 | HEX | *sodC* | *H. aegyptius* NCTC 8502 | - |
| D06 | HEX | *sodC* | *Moraxella catharalis* 03-0025-4 | - |
| E06 | HEX | *sodC* | *M. catharalis* 03-0028-5 | 38.72 |
| F06 | HEX | *sodC* | Water, Molecular Biology Grade | - |
| The assay was performed with a panel of 44 DNA samples and a no template control (Water, Molecular Biology Grade); Ct <35: positive result; Ct ≥ 35: negative result; -: negative result with Ct >50; NCTC, ATCC strains, *N. gonorrhée* WHO and *H. haemolyticus* QAF GSK were control strains, Oro means that the strain was isolated from oropharyngeal sample, Sal means that the strain was isolated from saliva sample. | | | | |
